# Unequal access to diagnosis of myalgic encephalomyelitis in England

**DOI:** 10.1186/s12889-025-22603-9

**Published:** 2025-04-22

**Authors:** Gemma Louise Samms, Chris P. Ponting

**Affiliations:** MRC Human Genetics Unit, The University of Edinburgh, Edinburgh, Scotland EH4 2XU UK

**Keywords:** Myalgic Encephalomyelitis prevalence, Ethnicity bias, NHS England, Gender bias

## Abstract

**Background:**

People with Myalgic Encephalomyelitis (ME/CFS; sometimes referred to as chronic fatigue syndrome) experience poor health-related quality of life and only rarely recover. ME/CFS has no curative treatment, and no single diagnostic test. Public health and policy decisions relevant to ME/CFS require knowledge of its prevalence and barriers to diagnosis. However, people with ME/CFS report lengthy diagnostic delays and prevalence estimates vary greatly due to uneven diagnosis and misdiagnosis. Factors that influence diagnosis could be revealed by stratifying a single population by gender, age and ethnicity.

**Methods:**

Hospital Episode Statistics data, routinely collected by the NHS in England, was downloaded from the Feasibility Self-Service of NHS DigiTrials. This was used to stratify individuals with the ICD-10 code that best reflects ME/CFS symptoms (G93.3) according to age, self-reported gender and ethnicity, General Practice and NHS England Integrated Care Board (ICB).

**Results:**

In all, 100,055 people in England had been diagnosed with ME/CFS (ICD-10:G93.3) between April 1 1989 and October 7 2023, 0.16% of all registered patients. Of these, 79,445 were females and 20,590 males, a female-to-male ratio of 3.88:1. Female relative to male prevalence peaked at about 6-to-1 in individuals’ fourth and fifth decades of life. Prevalence varied widely across the 42 ICBs: 0.086%-0.82% for females and 0.024%-0.21% for males. White individuals were approximately fivefold more likely to be diagnosed with ME/CFS than others; Black, Asian or Chinese ethnicities are associated with particularly low rates of ME/CFS diagnoses. This ethnicity bias is stronger than for other common diseases. Among active English GP practices, 176 (3%) had no registered ME/CFS patients. Eight ICBs (19%) each contained fewer than 8 other-than-white individuals with a G93.3 code despite their registers containing a total of 293,770 other-than-white patients.

**Conclusion:**

Other-than-white ethnic groups, older females (> 60y), older males (> 80y), and people living in areas of multiple deprivation are disproportionately undiagnosed with ME/CFS. Lifetime prevalence of ME/CFS for English females and males may be as high as 0.92% and 0.25%, respectively, or approximately 404,000 UK individuals overall (0.6%). This improved estimate of ME/CFS prevalence allows more accurate assessment of the socioeconomic and disease burden imposed by ME/CFS.

**Supplementary Information:**

The online version contains supplementary material available at 10.1186/s12889-025-22603-9.

## Background

At the population scale, estimates of disease morbidity and prevalence inform public health and policy decisions; for individuals, accurate diagnosis informs symptom management and treatment. Myalgic Encephalomyelitis (ME; sometimes referred to as Chronic Fatigue Syndrome [1]) is a chronic multi-system disorder resulting in very poor health-related quality of life and from which patients only rarely recover [2, 3]. ME/CFS is relatively common [4]: prevalence estimates in the UK or the USA range from 0.2%-0.4% [5–7], depending on case definition. A meta-analysis of 56 data sets covering 1.1 m individuals in 13 countries indicated an even higher prevalence, at 0.89% (95% CI: 0.60–1.33) [8]. However, this study’s criteria did not require post-exertional malaise (PEM), the worsening or new appearance of symptoms after previously tolerated physical or cognitive exertion, which nevertheless is a primary symptom of ME/CFS [9]. More recently, the USA National Health Interview Survey reported 1.3% of adults having a ME/CFS diagnosis and ongoing symptoms during 2021–2022 [10].

Historically, ME/CFS diagnosis in the UK has used various criteria, all with serious or very serious limitations [11, 12]. Revised diagnostic criteria were introduced in 2021 for use in NHS England and Wales [11, 12]. Uneven and incorrect application of these criteria results in about half of all cases referred to ME/CFS Specialist services not meeting diagnostic criteria [5, 13, 14]. ME/CFS diagnosis varies firstly by gender, with women diagnosed two- to five-times more often than men and also tending to report more severe symptoms [10, 15]. Diagnosis also may vary by ethnicity, although studies disagree on which group is more frequently diagnosed: African Americans and Native Americans [16], or White or Black (non-Hispanic) individuals [10], or African American and Latinx youth [7, 17].

In this study, we report Hospital Episode Statistics (HES) data for NHS Hospitals in England, specifically the ICD-10 code G93.3 (“Postviral fatigue syndrome”) which is the code that best reflects ME/CFS symptoms. Rather than being General Practice (GP) data, and rather than being UK-wide, the data is HES- and England-specific, and hence does not report UK-wide prevalence. Nevertheless, when stratified by age, self-reported ethnicity and gender, the data reveals substantial population variation in ME/CFS diagnosis.

## Methods

The NHS DigiTrials Feasibility Self-Service (England only [18]) returned all ME/CFS diagnoses since 1st April 1989, stratified by ICB, gender, age and self-reported ethnicity (accessed on 7–10 October 2023). For each stratum, NHS DigiTrials data is rounded: counts equal to or exceeding 8 are rounded to the nearest increment of 5; counts equal to or less than 7 were not provided and so were set to zero. Data was for 62,782,175 individuals who are alive, have a valid NHS number and are registered with a GP in England. Data also includes those who previously met these criteria but are no longer residing in England [19]. Individuals are recorded once without duplicates. NHS DigiTrials returns data derived from Hospital Episode Statistics (HES) for admitted patient care and outpatient appointments [19]. HES codes are either for the primary reason for the hospital visit or secondary/pre-existing conditions. HES are linked via the patients’ registered GP practice, and these 6,119 GP practices are linked to 42 ICBs. 6 GP practices were not considered further because they had fewer than 2,500 registered patients. HES data is updated monthly, on the second day of each month. Personal demographic data is updated daily. Patients with multiple diagnoses of the same type/code are counted only once, specifically the latest occurrence. As in other studies (e.g., [20]) we consider G93.3 to be the ICD-10 code that best reflects ME/CFS symptoms.

We apply the UK Government’s recommendations for describing ethnicity [21]. NHS DigiTrials uses self-reported ethnicity as classified in the 2001 census. ‘White’ includes 'white British', 'white Irish' and ‘Any other white background’. ‘Other than white’ includes any other ethnic group; Asian or Asian British—Any other Asian background; Asian or Asian British—Bangladeshi; Asian or Asian British—Indian; Asian or Asian British—Pakistani; Black or Black British—African; Black or Black British—Any other Black background; Black or Black British—Caribbean; Chinese; Mixed—Any other mixed background; Mixed—White & Asian; Mixed—White & Black African; Mixed—White & Black Caribbean). These two categories exclude those whose ancestry is ambiguous (i.e. 'not stated' or 'unknown’). Individuals whose ethnicity was 'not stated' or 'unknown’ were included for non-ethnicity related analyses.

NHS DigiTrials provided numbers of ME/CFS patients coded with G93.3 in active GP practices in England. For each GP practice these were indicated as between 1 and 7, or if equal to or above 8, provided to the nearest increment of 5. Active practices were those without a closed date, with ≥ 2,500 registered patients, whose status is ‘active’ and ‘operational’ (similarly for its related ICB), and with a valid postcode.

For Fig. 2B and C patients were partitioned into deciles of English indices of multiple deprivation (IMD; 2019) by their address (1 = most deprived, 10 = least deprived). For Fig. 4, English indices of deprivation for GP practices were acquired using their postcodes [22].

## Results

### Prevalence by age, deprivation and gender

**Fig. 1:**
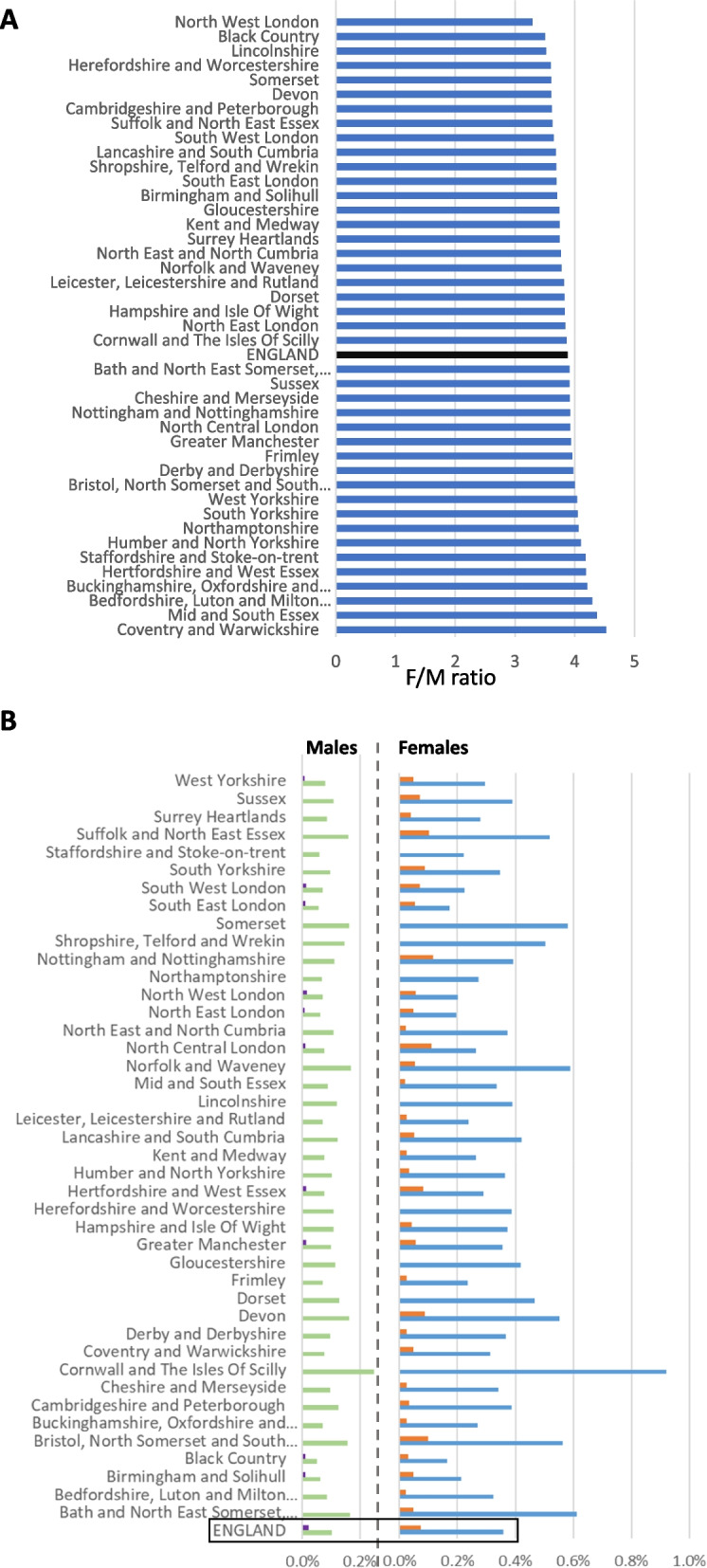
Diagnoses across 42 English Integrated Care Boards (ICB). **A** Ratios of female-to-male ME/CFS diagnoses (ICD-10:G93.3 codes) by ICB. **B** Point prevalence of males (left) or females (right) with ME/CFS stratified by ethnicity: either white or other-than-white. Blue: white females; Orange: Other-than-white Females; Green: white Males; Purple: Other-than-white Males

On 7 October 2023, 100,055 people had an electronic health record code for ME/CFS across the 42 NHS England Integrated Care Boards (ICBs). These are among 62,782,175 people (0.16%) with a valid NHS number, who were registered with a GP and were not deceased. This information was supplied by the Feasibility Self-Service of NHS DigiTrials [18] who provide Hospital Episode Statistics that are routinely collected by the NHS in England and held by NHS England. In all, 79,445 (0.25% of 31,297,675) females and 20,590 (0.065% of 31,481,510) males were associated with the ICD10 G93.3 code, a female-to-male (F/M) ratio of 3.88:1; 20 people with ME/CFS did not specify their gender. F/M ratio exceeded 3.3 across all of the 42 ICBs (range 3.3–4.5) (Fig. 1A).

Across the ICBs, female or male prevalence varied by an order of magnitude: 0.086%-0.82% and 0.024%-0.21%, respectively. Cornwall and the Isles of Scilly had the highest prevalence for each gender, and North West or North East London the lowest for females or males, respectively (Fig. 1B; Additional file 1).

**Fig. 2:**
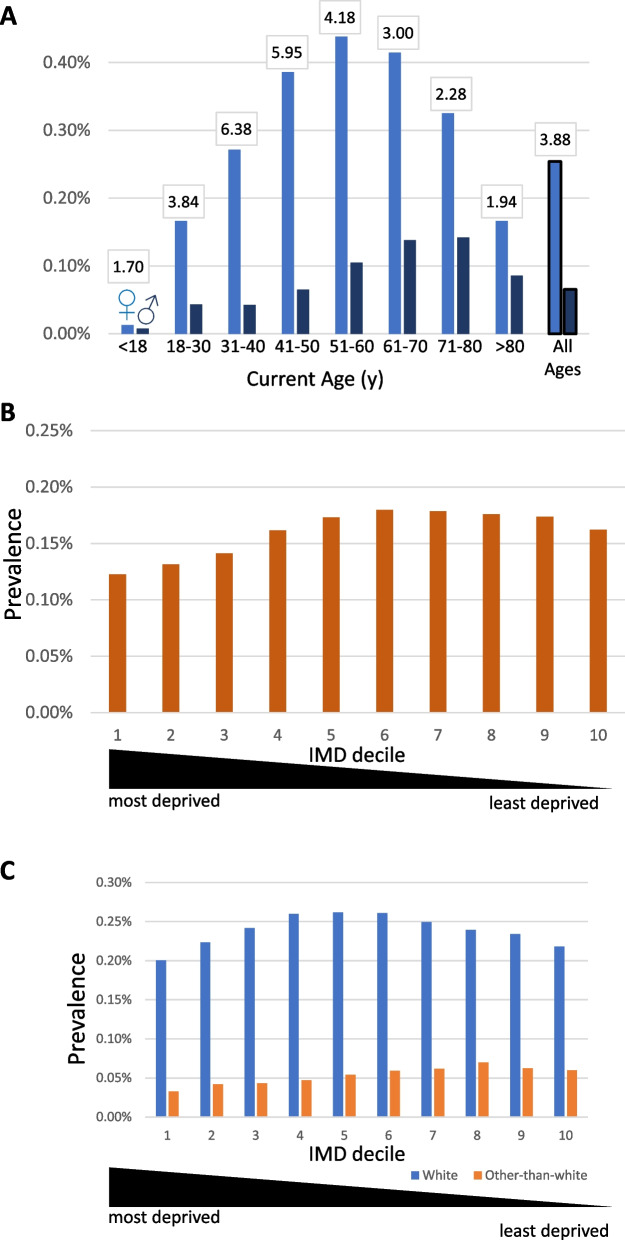
ME/CFS diagnoses by age or deprivation. **A** Ages of females or males with ME/CFS diagnoses in England (light blue: females; dark blue: males; black: overall ratio) as of 7th October 2023. The female-to-male ME/CFS diagnosis ratios for every age decade are shown in boxes. (B) ME/CFS patients partitioned by Indices of multiple deprivation (IMD) in deciles. (**C**) As (**B**), but separated by self-reported white or other-than-white ethnicities

Point prevalence of ME/CFS varied greatly across lifespan, peaking at about 50 years of age for females and over a decade later for males (Fig. 2A). In individuals’ fourth and fifth decades the F/M ratio peaked at an exceptionally large value, approximately 6-to-1.

ME/CFS prevalence varied 1.5-fold by deprivation assessed by the Index of Multiple Deprivation (IMD). The IMD is a measure of relative deprivation for small areas in England [22] combining seven domains: income; employment; education, skills and training; health and disability; crime; housing and services; and the living environment. Each small area has been ranked and divided into tenths (‘deciles’) from the most deprived (decile 1) to the least deprived (decile 10). ME/CFS prevalence was lowest for those living in areas among the three most deprived deciles (Fig. 2B).

### Prevalence by ethnicity

Ethnic groups showed unexpectedly large variation in ME/CFS prevalence. Specifically, white prevalence was 4.9-fold higher than other-than-white prevalence (0.24% vs 0.049%, respectively). This finding was observed across all ICBs for both females and males (Fig. 1B). Variation of ME/CFS prevalence by IMD deciles was similar between white and other-than-white ethnicities (Fig. 2C).

**Fig. 3:**
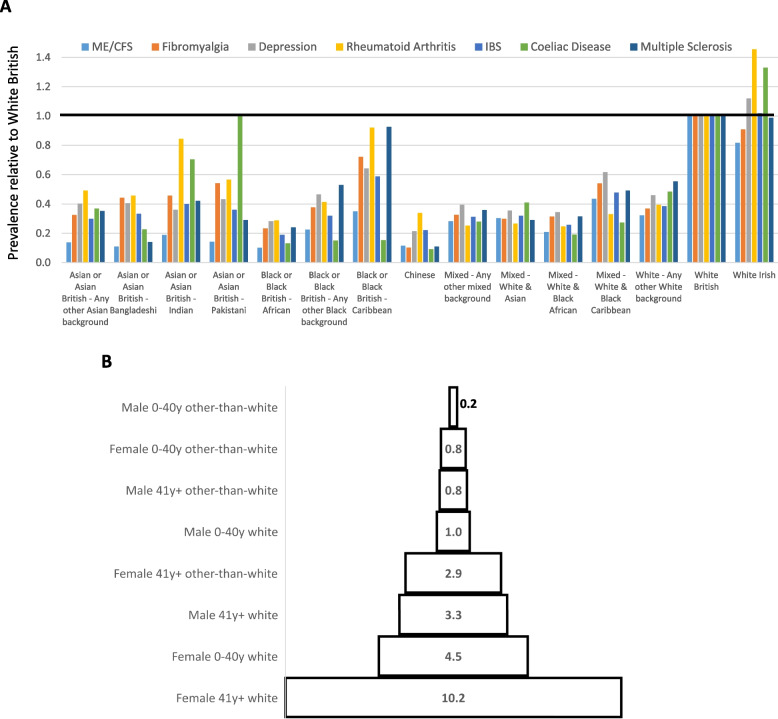
Relative prevalence of ME/CFS and other diseases stratified by ethnic group, age and gender. **A** Prevalence of ME/CFS (ICD-10:G93.3), Fibromyalgia (ICD-10: M79.7), Rheumatoid arthritis (M05.3, M05.8, M05.9, M06.0, M06.8, M06.9 or M08.0), clinical depression (F32 or F33 codes), Irritable Bowel Syndrome (K58.0 or K58.9), Coeliac Disease (K90.0), or Multiple Sclerosis (G35) for self-reported ethnicities relative to ‘white British’. Codes were downloaded from NHS DigiTrials on 8^th^ October 2023. **B** ME/CFS prevalence for female or male groups, of younger or older age, and of white or other-than-white ethnicity, relative to young (≤ 40y old) white males (normalised to 1.0)

ME/CFS prevalence of other-than-white British individuals was between one-tenth and one-third that for white British. Those with Chinese, Asian/Asian British or Black/Black British ethnicity were 11%, 11%-19%, or 10%-35%, respectively, less likely to be diagnosed with ME/CFS (Fig. 3A). The low prevalence of ME/CFS for other-than-white ethnicity categories is more profound than for other diseases, for example fibromyalgia and clinical depression (Fig. 3A).

Eight ICBs each contained fewer than 8 other-than-white individuals with a G93.3 code (≤ 0.0027% for 293,770 other-than-white individuals). These 8 ICBs were Dorset; Gloucestershire; Herefordshire and Worcestershire; Lincolnshire; Northamptonshire; Shropshire, Telford and Wrekin; Somerset; and, Staffordshire and Stoke-on-Trent.

In summary, ME/CFS diagnosis in England is highest among older, white females, and lowest among younger other-than-white males; the variation is 50-fold between these three categories (Fig. 3B).

### Prevalence per GP practice

**Fig. 4:**
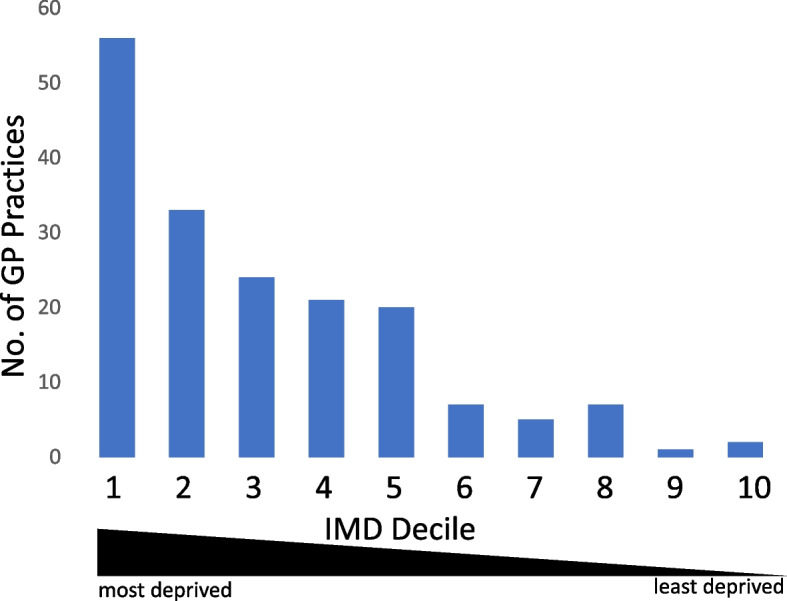
English GP practices. Numbers of active GP practices without registered ME/CFS patients recorded in HES stratified by indices of multiple deprivation

Lastly, we considered 6,113 English GP practices with at least 2,500 registered patients which each, given the national prevalence of 0.16% (above), would be expected to have 4 or more ME/CFS patients registered. Two-thirds of these practices (4,061; 66%) had 8 or more registered patients with ME/CFS and 97% (5,937) reported at least one individual with ME/CFS. Of the remaining practices, 176 (3%) had no registered ME/CFS patients recorded in HES despite being operational and having a median of 4,765 registered patients (range 2,665–12,170), a total population of 917,570. These 176 practices are disproportionately located in the most deprived areas of England (Fig. 4).

## Discussion

Across England, ME/CFS diagnosis varies 50-fold, with considerable variation by gender, age, ethnicity, and location (Figs. 1, 2C and 3B. Female-bias contributes about fourfold, mid-way among previous values for single-country or international ME/CFS cohorts (1.5–sixfold [1, 15, 20, 23–26]). This wide variation may result, in part, from cohorts sampling from different age ranges (Fig. 2A). Young (< 40y) or old (> 40y) age contributes ~ threefold to ME/CFS prevalence variation (Figs. 2A; 3B), reflecting an accumulation of ME/CFS diagnoses over adult life. Decreased ME/CFS female prevalence in later life (> 60y; Fig. 1B) cannot be due to recovery, as records are deleted only rarely, or increased mortality [27] but could reflect historically low levels of diagnoses. The 20y difference between the highest male (ages 71-80y) and highest female (ages 51-60y) prevalence is puzzling. It may, however, reflect gender-specific historical variation in ME/CFS diagnosis and/or hospital attendances peaking 40y earlier for females than for males [28]. Older females (> 60y) and males (> 80y) are less likely to be coded with ME/CFS in their hospital records (Fig. 2A). However, note that if they were diagnosed before 1st April 1989 and were not since re-coded in their hospital records, their diagnosis will be missing from the data we analysed.

White versus other-than-white ethnicity accounts for a further ~ fivefold variation (Fig. 3B), with some ethnicities (e.g. Black, Asian or Chinese ancestry) associated with particularly low rates of ME/CFS diagnoses (Fig. 3A). This ethnicity bias is stronger than for other common diseases such as dementia, ischaemic heart disease, clinical depression, and fibromyalgia [29] (Fig. 3A). The bias is unlikely to reflect genetic differences [30], or to unequal access to primary care because other-than-white individuals are not less likely to visit GP services [31]. Rather it is likely due to fewer hospital visits by people of East Asian ancestry [31] and to wider social and economical health factors [32]. Barriers to diagnosing and managing ME/CFS in other-than-white groups have previously been recognised [33]. These will need to be overcome if there is to be equitable access to ME/CFS diagnosis and healthcare.

Age-at-ME/CFS diagnosis is highly variable, especially for paediatric ME/CFS cases: this study identified few < 18y old ME/CFS cases in England (0.010% for both genders combined; Fig. 2A), consistent with the UK overall [15]. In other countries, however, paediatric prevalence of ME/CFS is much higher [8], e.g., 0.75% in a USA community cohort [17]; 26% of Norwegian ME/CFS patients were younger than 20y old [20]. Paediatric ME/CFS referral services in England and the rest of the UK are limited [34]. Most of the 100,055 people analysed in this study will have acquired ME/CFS symptoms prior to the COVID-19 pandemic and so include few of the many Long Covid-19 patients whose symptoms meet ME/CFS diagnostic criteria [35] and who nevertheless are rarely diagnosed with ME/CFS [15].

A strength of this study is that it captures all ME/CFS diagnoses given the G93.3 ICD-10 code during admissions and outpatient appointments at English NHS hospitals up to October 2023. It is over two orders of magnitude larger in scale than a primary care study of 3 English areas [5] whose prevalence estimate (0.11–0.20%) is comparable to this study’s G93.3 prevalence (0.16%). It is more up-to-date than a 2008–2010 study of diagnosis rates covering only English specialist ME/CFS services [36]. A weakness is that those whose ME/CFS was diagnosed in primary care will only have been included if their G93.3 code was subsequently added to the HES data in secondary care. Primary care diagnoses of ME/CFS left unrecorded in HES data will under-estimate ME/CFS prevalence. This under-estimation could be substantial: among UK Biobank (UKB) participants with a linked ME/CFS code in primary care records and who also have available hospital inpatient data, only 28% are linked to a G93.3 code [37]. This may be due to missing or asynchronous data, but also to UKB HES data being linked to only inpatient, not outpatient, appointments, and to G93.3 not being applied to approximately one-third [15] of patients whose ME/CFS symptoms were not triggered by a viral infection. The G93.3 code does not greatly over-estimate UKB cases because most (72%) have further evidence of ME/CFS [37].

With this study’s data, we can now estimate the number of people in the UK who would have a ME/CFS diagnosis (i.e. the G93.3 code) if there were minimal social and healthcare barriers to diagnosis. For this, we used the maximal ME/CFS prevalences for white females and males (0.92% and 0.25%, respectively), which are for NHS Cornwall and Isles of Scilly (Fig. 1B). This ICB would be expected to have the highest fraction of diagnosed ME/CFS patients as it has the oldest population (average ~ 45y [females] and ~ 44y [males]), the highest F/M ratio (1.04) and, most strikingly, the lowest other-than-white population (2.0%). If all 68.3 million UK citizens [38] were to have a lifetime prevalence matching the point prevalence of NHS Cornwall and Isles of Scilly then 83,626 males (i.e., 0.25% of 33.450m) and 320,296 females (i.e., 0.92% of 34.815m) would be given a G93.3 ME/CFS diagnosis in their lifetime. These are lower bound estimates because no account is taken of those in NHS Cornwall and Isles of Scilly who have yet to receive a ME/CFS diagnosis. This total of 403,922 would be a 62% increase over a current prevalence estimate of 250,000 [11]. Even if half of these do not meet more stringent diagnostic criteria for ME/CFS (as previously [5, 14], then this reduction could be offset by those diagnosed in primary care or private healthcare, but not coded in the HES data analysed in this study including because their ME/CFS was not triggered by a viral infection. In summary, we suggest that the 2023 UK lifetime prevalence of ME/CFS diagnosed using stringent criteria is approximately 320,000 for females and 84,000 for males (i.e., 0.92% and 0.25%, respectively).

## Conclusions

These results reveal deficiencies in ME/CFS diagnosis across different groups in England. To address these deficiencies, improved training of medical professionals should be available [39] and research into identifying accurate diagnostic tests should be prioritised. Even when diagnosed, there is no curative therapy for ME/CFS, only symptom management [39]. Nevertheless, an individual’s ME/CFS diagnosis provides considerable value as it: (i) counters the delegitimization of problems often experienced by ME/CFS patients [40, 41], (ii) improves patient/professional relationships, (iii) facilitates symptom management, (iv) enables application for disability benefits, (v) assists recruitment to future clinical trials of potentially effective treatments, and (vi) helps individuals to join supportive and informative patient communities.

## Supplementary Information

Additional file 1. NHS DigiTrials Feasibility Self-Service data on ME/CFS diagnoses since 1^st^ April 1989, stratified by ICB, gender, age and self-reported ethnicity (.xlsx format).

## Data Availability

NHS data is copyright © 2022, NHS England, re-used with the permission of NHS England. All rights reserved. Access to this data was provided by NHS DigiTrials and the Feasibility Self-Service. NHS DigiTrials Feasibility Self-Service data on ME/CFS diagnoses since 1st April 1989, stratified by ICB, sex, age and self-reported ethnicity is available as Additional file 1 (.xlsx format).

## Abbreviations

CI: Confidence interval
F/M: Female-to-male
GP: General Practice
HES: Hospital Episode Statistics
ICB: NHS England Integrated Care Board
ICD10: International Classification of Diseases 10th Revision
IMD: Indices of multiple deprivation
ME/CFS: Myalgic Encephalomyelitis / Chronic Fatigue Syndrome
NHS: National Health Service
